# 

**Published:** 1881-02

**Authors:** 


### CONTRIBUTORS.

| | |
|---|---|
| Professor H. C. Allen, - - | Ann Arbor. |
| " T. F. Allen, - - | New York City. |
| Doctor Edward Bayard, - - | " " " |
| " J. B. Bell, - - - - - - | Boston. |
| " E. W. Berridge, - - - - | London. |
| " Ad. Fellger, - - - - | Phila. |
| " John Hall, - - - | Toronto, Canada. |
| " W. H. Jenny, - - | Kansas City, Mo. |
| " Ad. Lippe, - - - - - | Phila. |
| " C. Lippe, - - - | New York City. |
| " M. MacFarlan, - - - - | Phila. |
| " Thos. Moore, - - - - | " |
| " C. F. Nichols, - - - | Boston. |
| " C. Pearson, - - - | Washington. |
| " Thomas Skinner, - - | Liverpool. |
| " S. Swan, - - - - | New York. |
| " P. P. Wells. - - - | Brooklyn. |
| " Wm. Wesselhoeft, - - - | Boston. |
| Professor T. P. Wilson, - - | Ann Arbor. |

&c. &c. &c.

N. B. Authors of accepted papers are furnished with copies of the number in which their articles appear; application for such copies to be made when sending papers.

A prize of fifty dollars will be awarded to author of the best essay.

